# Effects of immersive virtual reality for preventing and managing anxiety, nausea and vomiting among paediatric cancer patients receiving their first chemotherapy: A study protocol for an exploratory trial

**DOI:** 10.1371/journal.pone.0258514

**Published:** 2021-10-14

**Authors:** Cho Lee Wong, Chi Kong Li, Kai Chow Choi, Winnie Kwok Wei So, Jojo Yan Yan Kwok, Yin Ting Cheung, Carmen Wing Han Chan

**Affiliations:** 1 The Nethersole School of Nursing, Faculty of Medicine, The Chinese University of Hong Kong, Hong Kong, Hong Kong; 2 Department of Paediatrics, Faculty of Medicine, The Chinese University of Hong Kong, Hong Kong, Hong Kong; 3 School of Nursing, The University of Hong Kong, Hong Kong, Hong Kong; 4 School of Pharmacy, Faculty of Medicine, The Chinese University of Hong Kong, Hong Kong, Hong Kong; Public Library of Science, UNITED KINGDOM

## Abstract

**Background:**

Anxiety, nausea and vomiting are common side effects suffered by paediatric patients receiving chemotherapy. Emerging evidence supports the efficacy of immersive virtual reality (IVR) on improving anxiety and distress symptoms including nausea and vomiting among this vulnerable group. This trial aims to assess the feasibility and acceptability of IVR for preventing and managing anxiety, nausea and vomiting among paediatric cancer patients receiving their first chemotherapy.

**Method and analysis:**

An exploratory trial supplemented by qualitative methods will be conducted. We will recruit 20 paediatric patients who are aged between 6 and 12 years, chemotherapy naïve, scheduled to receive their first intravenous chemotherapy and able to understand Chinese. Participants will be randomly allocated to intervention or control groups. The intervention group will receive the IVR intervention for three sessions as follows: 4 hours before chemotherapy, 5 minutes before and during their first course chemotherapy and 5 minutes before and during their second course chemotherapy. The control group will receive standard care only. Main outcome measures included (1) key parameters for the design of a definitive trial (i.e. screening, eligibility, consent and withdrawal rates); (2) anxiety, anticipatory and acute chemotherapy-induced nausea and vomiting for collection of preliminary data; (3) feasibility and acceptability of the intervention. Semi-structured interviews will be conducted with patients, parents and oncology nurses. Generalized estimating equations model will be used to compare each of the outcome measures across the time points between the two groups. Qualitative data will be analysed by conventional content analysis.

**Expected results:**

The results of this exploratory trial will inform the design and conduct of future definitive trial.

**Trial registration number:**

ChiCTR1900021694; Pre-results.

## Introduction

Chemotherapy treatment can provoke anxiety in many patients [1]. In particular, patients usually exhibit a heightened level of anxiety when receiving their first chemotherapy [2]. An observational study found that 89% of chemotherapy-naïve patients experienced pre-chemotherapy anxiety. Paediatric cancer patients exhibiting elevated anxiety level early in the therapy are four times more prone to have elevated anxiety after the treatment [1]. Moreover, pre-chemotherapy anxiety, as well as anxiety experienced after chemotherapy start, expose patients to a higher risk of chemotherapy-induced nausea and vomiting (CINV) [3, 4].

CINV remains the most frequent and unpleasant side effect in paediatric patients receiving chemotherapy [5]. Acute CINV refers to symptoms that occur within 24 hours of chemotherapy administration [5]. Anticipatory CINV may develop without proper initial management. Anticipatory CINV is a conditioned response that occurs prior to a subsequent initiation of chemotherapy upon being re-exposed to the stimuli that signal the chemotherapy infusion [5]. A meta-analysis of 35 studies reveal that 30% of adult and paediatric patients reported anticipatory CINV [6]. One known factor associated with CINV development among paediatric patients is emetogenicity of chemotherapy [7]. However, pharmacological treatments for CINV prevention are still suboptimal [5].

Poorly managed anxiety and CINV lead to physical consequences, such as dehydration and electrolyte imbalance, and hinder the patients’ ability to cope with subsequent chemotherapy. This phenomenon may lead to the delay or discontinuation of treatment and affect treatment and survival outcomes [7]. CINV is the most aversive condition that greatly distresses patients and parents, thereby reducing their satisfaction to care [7].

Effective management of anxiety and CINV is critical for children receiving their first chemotherapy; in particular, anticipatory and acute CINV increase with time [6]. International guidelines recommend optimal psychological interventions be offered to children for anticipatory CINV [8]. Therefore, psychological interventions are needed to help paediatric cancer patients to cope with anxiety and CINV on their first chemotherapy.

Distraction is among the most effective psychological interventions in reducing anxiety and distress in paediatric patients [9]. A systematic review and meta-analysis in paediatric patients show that distraction interventions without adult involvement and use of interactive form are more effective in reducing distress, especially for patients younger than 12 years old [9]. Previous studies also demonstrated that distraction and relaxation are useful for paediatric patients undergoing medical procedures [10, 11]. However, face-to-face distractions and relaxation interventions required adult involvement and intensive labour. Therefore, other cost-effective and interactive options should be considered.

Virtual reality (VR) is an interactive form of distraction wherein a human becomes an active participant in a virtual environment [12]. VR ranges from non-immersive to fully immersive depending on the degree of the user’s isolation from physical surroundings. In immersive VR (IVR), immersion is created through a head-mounted display which allows 3D interaction between the user and computer. Compared with non-immersive VR, IVR has advantages, including less specialized facilities required and provision of an immersive environment that completely turns the participants’ attention away from the noxious clinical environment [12]. The cost of IVR is also becoming increasingly affordable [12]. For example, the price of a commercially available disposable IVR headset (Google Cardboard Goggles) is HK $20 (~USD$2.57). Distraction-based IVR intervention is increasing used in paediatric setting [13–15].

A recent review suggested that IVR effectively reduces anxiety and distress for adults and paediatric patients immediately after chemotherapy sessions [12]. In addition, the efficacy of IVR does not decrease after 8 weeks of repeated exposures in a controlled laboratory environment [16]. IVR also helped patients to alter their perception of time, thus making the chemotherapy treatment seem short [17, 18]. A pilot study on 11 paediatric patients (aged 10–17) suggested that IVR can be implemented and positively accepted [19]. Another pre- and post-test study revealed that children receiving chemotherapy reported improved anxiety and distress symptoms, including nausea and vomiting, after one session of IVR intervention [2]. Nevertheless, these findings are limited by methodological weaknesses, such as a lack of control group or pre-test evaluation or a lack of follow up [17–19]. In addition, the assessment of paediatric patients, parents and health care providers’ perception and satisfaction towards IVR intervention is seldom included [12].

To our knowledge, no previous work is conducted to examine the effects of IVR on paediatric cancer patients undergoing chemotherapy in Hong Kong. The Medical Research Council presented a framework for the evaluation of a complex intervention and recommended a stepwise approach to evaluation with exploratory trial preceding a full randomized controlled trial [20]. As such, an innovative trial is proposed for the assessment of the effect of IVR to establish relaxation as counter conditioning and distraction in the intervention package.

### Aim and objectives

This exploratory trial aims to assess the feasibility and acceptability of IVR as a relaxation and distraction intervention for reducing anxiety and CINV among paediatric cancer patients receiving their first chemotherapy.

1. The objectives of this trial are:
2. To assess the parameters and feasibility for the design of a definitive trial, including screening, eligibility, consent and withdrawal rates.
3. To assess the data collection procedure and collection of preliminary data including anxiety (self-reported and physiological responses including heart rate and mean arterial blood pressure), anticipatory and acute CINV between the intervention and control groups.
4. To assess the satisfaction of the chemotherapy procedure to parents and nurses.
5. To explore ways of improvement for the implementation of intervention and the acceptability of the intervention to patients, parents and nurses.

## Methods and analysis

### Design

This is an exploratory trial supplemented with qualitative methods. The protocol is registered on Chinese Clinical Trial Registry (ChiCTR1900021694, March 5, 2019). Participants will be randomly allocated to an intervention group that will receive the IVR intervention or the control group.

### Participants

Patients and their parents, as well as oncology nurses will be recruited from the oncology unit of a paediatric hospital.

#### Patients

Patients will be included if they are/ can (1) aged between 6 and 12 years, (2) chemotherapy naïve, (3) scheduled to receive their first intravenous chemotherapy and (4) understand Chinese. Those who have (1) identified cognitive and learning problems in their medical record, (2) brain tumours or metastasis, (3) identified contact precautions and (4) previous history of seizures or motion sickness will be excluded.

The rationale for selecting patients aged 6–12 years is because younger patients are more susceptible to anxiety when undergoing medical procedures as compared with older ones. Therefore, their cooperation and response to the chemotherapy procedure and intervention are of concern [10, 11, 15]. Meanwhile, this homogenous age group undergoes the same concrete operational developmental stages and thus is more responsive to distraction and relaxation intervention [9, 11].

#### Accompanying parents

All accompany parents will be invited to assess their satisfaction for the chemotherapy procedures. Parents in the intervention group will also be invited to a semi-structured interview to explore their acceptability of the intervention.

#### Oncology nurses

All oncology nurses involved in administration of chemotherapy will be invited to assess their satisfaction for the chemotherapy procedures. Nurses in the intervention group will also be invited to semi-structured interviews to explore their perceptions of trial procedures and the feasibility of integrating the intervention into routine clinical practice.

### Sample size calculation

In 2017, about 28 paediatric cancer patients aged between 6 and 12 years were admitted to the study institution for their first chemotherapy. This exploratory trial recruits 20 patients, 20 accompany parents and 10 oncology nurses involved in the administration of chemotherapy. Based on our previous trial on paediatric patients with cancer, this sample size can be realistically recruited within the study period and is adequate for the objectives of the trial [11, 15].

### Randomisation and blinding

This exploratory trial comprises two groups: an intervention group that receives the IVR intervention and standard care and a control group that receives standard care only. A computer-based stratified randomization is used to randomly assign patients into one group or the other. Strata are constructed on the basis of cancer types, approach commonly adopted in previous studies [6, 11]. Randomization is conducted by an independent statistician.

The nature of an IVR intervention may be impractical for the blinding of participants and interventionists. Nevertheless, the lack of blinding does not necessarily contribute to a source of bias because children are unlikely to change their behaviours even when they are aware of participating in a certain intervention [10, 11].

### Control: Standard care

Participants in the control group will receive standard care only with no IVR intervention during their chemotherapy. Standard care consists of pre-chemotherapy instruction and intravenous antiemetic administration as needed.

### Intervention: IVR

Participants receive IVR for three separate sessions in the first and second course of chemotherapy: (1) 4 hours prior to the chemotherapy commenced, (2) 5 minutes before and during the first chemotherapy and (3) 5 minutes before and during the second chemotherapy. Considering additional sessions may result in missing data despite enhancing the intervention effect, therefore, three sessions of IVR appears feasible [21].

The first IVR session is used to establish a relaxation and playful response to participants before the chemotherapy commenced. As patients are usually admitted in the morning to prepare for chemotherapy infusion that is commenced in the afternoon, the first IVR session is held 4 hours before commencement of chemotherapy for practical reason. The second IVR session is used to establish IVR as counter conditioning and distraction intervention during chemotherapy infusion to reduce the anxiety and CINV of patients. The third session aims to determine if IVR has stable effects on reducing anxiety and CINV, and whether or not patients can sustain their interest in IVR.

During the intervention, the patients experience the sense of immersion through a head-mounted device delivering IVR sounds and images by using disposable Google cardboard goggles that can be fitted to both Apple and Samsung smartphones. Latest headsets (Oculus Rift or HTC Vive) may increase contact infection risk and therefore not used in this trial.

Our previous work on IVR among paediatric cancer patients found that three-dimensional cartoon videos, such as Minions or Cut the rope, are preferred by patients aged below 12; however, VR sceneries are not selected by any children. As such, four 3D cartoon modules, which provide a wide range of visual and auditory stimuli and can be freely downloaded, are provided for the patients. These modules are considered as appropriate after consultation with oncologists, nurses in paediatric cancer units and an IT specialist. These videos are also interesting and captures the attention of patients for an extended period of time [15].

### Implementation protocol

A trained research assistant (RA) provides the patients with standard instruction on how to use the IVR equipment in the first session. The Google cardboard goggles will then be placed and adjusted on the patient’s head to ensure comfort and secure fit. For the first session, patients are given 30 minutes to use and familiarise themselves with the IVR equipment [17, 18].

For the second and third sessions, the RA provides IVR to patients 5 minutes before the chemotherapy commences [2, 19]. The RA asks the oncology nurse to start the administration once the patients are ready for the chemotherapy treatment. The nurse first disinfects outlets of the intravenous site, connect the device and start infusion, during which patients will be allowed to view their selected VR modules or take off the headset at will. The RA will record the time of each module used by the patients by using a time-counting device. When the infusion is over, the RA will remove the IVR equipment.

### Fidelity of the intervention

The fidelity of the intervention is ensured by recruiting a part-time RA who has at least two years of experience in paediatric care. The investigator will deliver 1.5 days of training to RA in (1) evidence of the benefits of IVR to paediatric patients; (2) common chemotherapy regimens; (3) procedures of implementing the intervention; and (4) application and basic knowledge about IVR. At least one session conducted by the RA each month is randomly selected to assess compliance with the implementation protocol by the principal investigator.

### Measurements

The following data will be collected:

- *Parameters and feasibility for designing a definitive trial*
  - Screening rate: The number of patients admitted to the paediatric oncology unit and screened for eligibility by the oncology nurse.
  - Eligibility rate: The number of patients fulfilling the inclusion criteria divided by the number of patients admitted to the unit for their first chemotherapy.
  - Consent rate: The number of patients whose parents give consent divided by the number of patients eligible for the trial. Those who do not give consent will have their reasons for declining (provided in voluntary basis) as recorded by the RA.
  - Withdrawal rate: The withdrawal rate refers to the number of patients withdrawing from the trial after giving consent. For ethical reasons, the number of participants who decline to participate in the project will only be recorded instead of asking them for the reason of their refusal to participate. However, such data will be recorded down if the patients provided in voluntary basis.
- *Quantitative Outcomes*
  - Anxiety: The short form of the Chinese version of the State Anxiety Scale for Children (CSAS-C) will be used to measure the self-reported anxiety levels of the participants in the trial [22]. This measurement uses a three-point Likert scale with 10 items and total scores ranging from 10 to 30, with higher scores indicating higher anxiety levels [22]. The State Anxiety Scale has been used in our previous IVR trial to measure anxiety among children undergoing needle-related procedures, with a Cronbach’s alpha coefficient of 0.89 [15].
  - Heart rate and mean arterial blood pressure: The physiological responses of anxiety will be measured by heart rate and mean arterial blood pressure (HR & BP) using a standard automatic blood pressure monitoring machine (available in the study institution). These indicators are considered to be objective and definitive in assessing physiological responses of anxiety [10, 11].
  - Anticipatory nausea and vomiting: A visual analogue scale (VAS) will be used to assess the severity of nausea and vomiting before the chemotherapy. Children will be asked to indicate their severity of nausea and vomiting on a 0–100 mm horizontal line, with “0” indicating the absence of nausea and vomiting while “100” indicates the most severe form of nausea and vomiting.
  - Acute nausea and vomiting: The MASCC Antiemesis Tool (MAT) will be adopted to assess the chemotherapy-induced nausea and vomiting. The Chinese version has good reliability and validity with a Cronbach’s alpha coefficient of 0.73 [23]. The four items assessing acute CINV will be used in this trial.
- *Satisfaction*
  The parent and nurse satisfaction levels toward the chemotherapy procedure will be assessed by questionnaires developed by Tyson and colleagues [24]. The original English questionnaire for parents is a 10-item scale rated on a five-point scale ranging from 1 = strongly disagree to 5 = strongly agree. Higher scores indicate higher levels of satisfaction. The satisfaction of the nurses will be examined by eight items, with each being rated on a scale from 1 = strongly disagree to 5 = strongly agree. The first author (CLW) has translated both questionnaires into Chinese using the back-translation method [25, 26]. The translated version was reviewed by a panel of expert professionals for semantic and content equivalence. This scale has been used in previous trial to assess the satisfaction level of parents and health care professionals toward a medical procedure with the reported Cronbach’s alpha 0.90 for both scales [10].
- *Acceptability of the intervention*
  Individual face-to-face semi-structured interviews will be conducted using an interview guide with 10 patients, their accompanying parents and the nurses who administrate chemotherapy to the intervention group. The interviews with the patients aim to explore their experiences, acceptance, satisfaction and any improvements of the IVR intervention. The interviews with parents aim to explore their acceptance and possible improvements in implementing IVR for their child. The interviews with nurses aim to determine the feasibility of integrating IVR intervention into their routine clinical practice and any possible improvements. The RA, who also has considerable experience in qualitative research, will conduct the interviews. Each interview will be audio-taped and conducted in a quiet room provided at the study institution.
- *Socio-demographic and clinical characteristics*
  The following clinical characteristics will be collected from participant’s medical record: age, gender, year of study, medical diagnosis, time since diagnosis, stage of disease, type of cancer treatment received, chemotherapy regimen, types and dosage of antiemetic prescribed and intake. These will be useful in providing baseline information for optimizing design and subject recruitment for the future definitive trial.

### Procedures

A nurse in the paediatric oncology unit will screen for eligibility for all patients who are admitted to receive their first intravenous chemotherapy during pre-chemotherapy assessment. If children meet the inclusion criteria, the nurse will refer them and their accompanying parents to the RA who will give them an information sheet, explain the trial and show the IVR equipment. If they agree to participate, an informed consent will be obtained from the accompanying parents. The RA will then acquire socio-demographics and clinical characteristics of the patients from medical record. According to the subject allocation, children in the control group will receive standard care, while the intervention group will additionally receive the IVR intervention.

**Fig 1:**
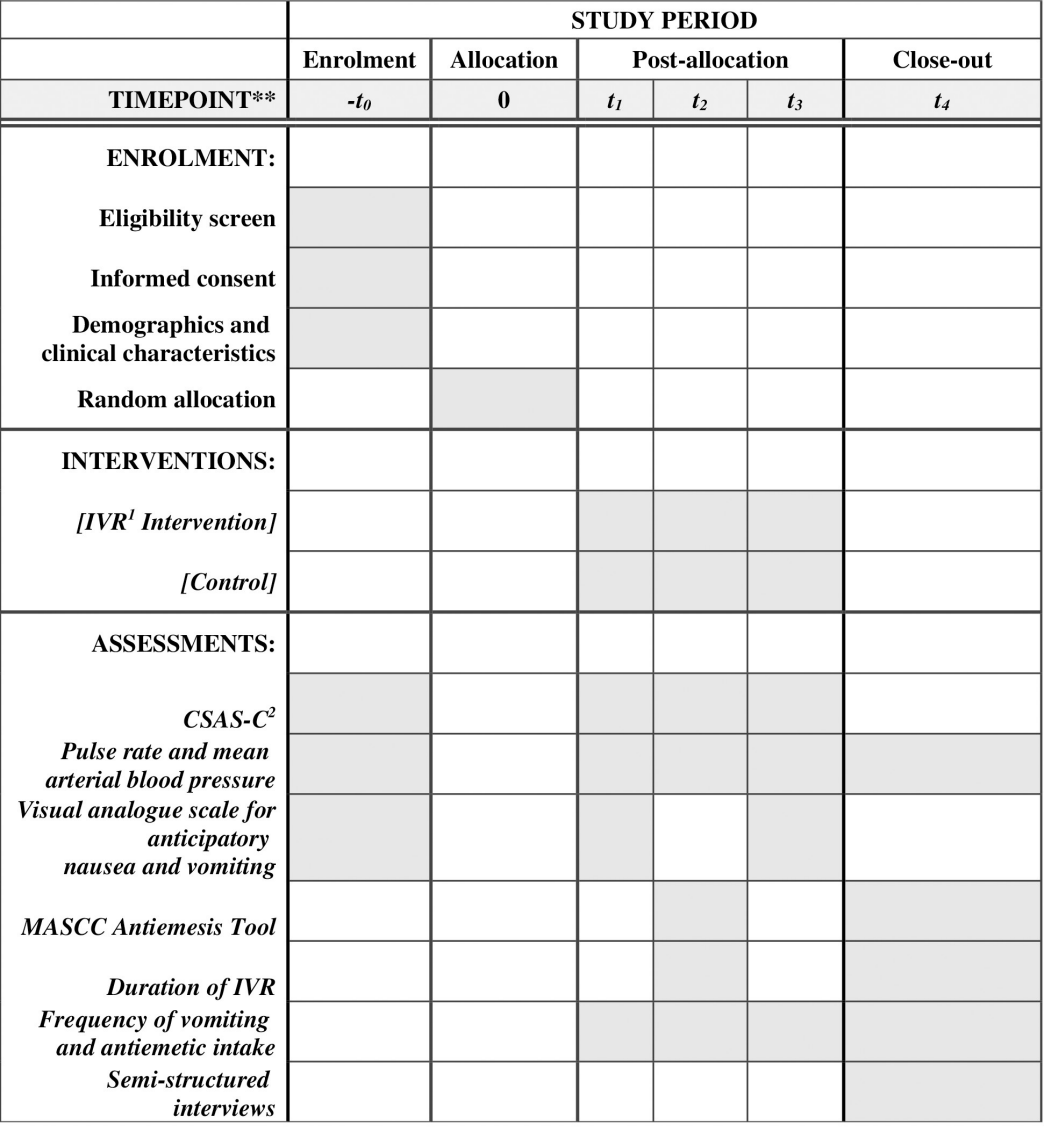
SPIRIT schedule of the trial. T0: Pre-test data 4 hours prior to chemotherapy; T1: Immediate before 1^st^ chemotherapy starts; T2: Immediate after 1^st^ chemotherapy; T3: Immediate before 2^nd^ chemotherapy starts; T4: Immediate after 2^nd^ chemotherapy. ^1^IVR: Immersive virtual reality; ^2^CSAS-C: The short form of the Chinese version of the State Anxiety Scale for Children’.

Four hours prior to (T0), immediate before first course (T1), and second course of chemotherapy begins (T3), a set of data (i.e. anxiety, HR & BP and anticipatory CINV) will be collected from the patients. Immediate after the first (T2) and second course of chemotherapy (T4), the anxiety, HR & BP and acute CINV will be collected. In the intervention group, the timing and duration of IVR use and modules selected will also be recorded. The SPIRIT schedule of the trial is shown in Fig 1.

**Fig 2:**
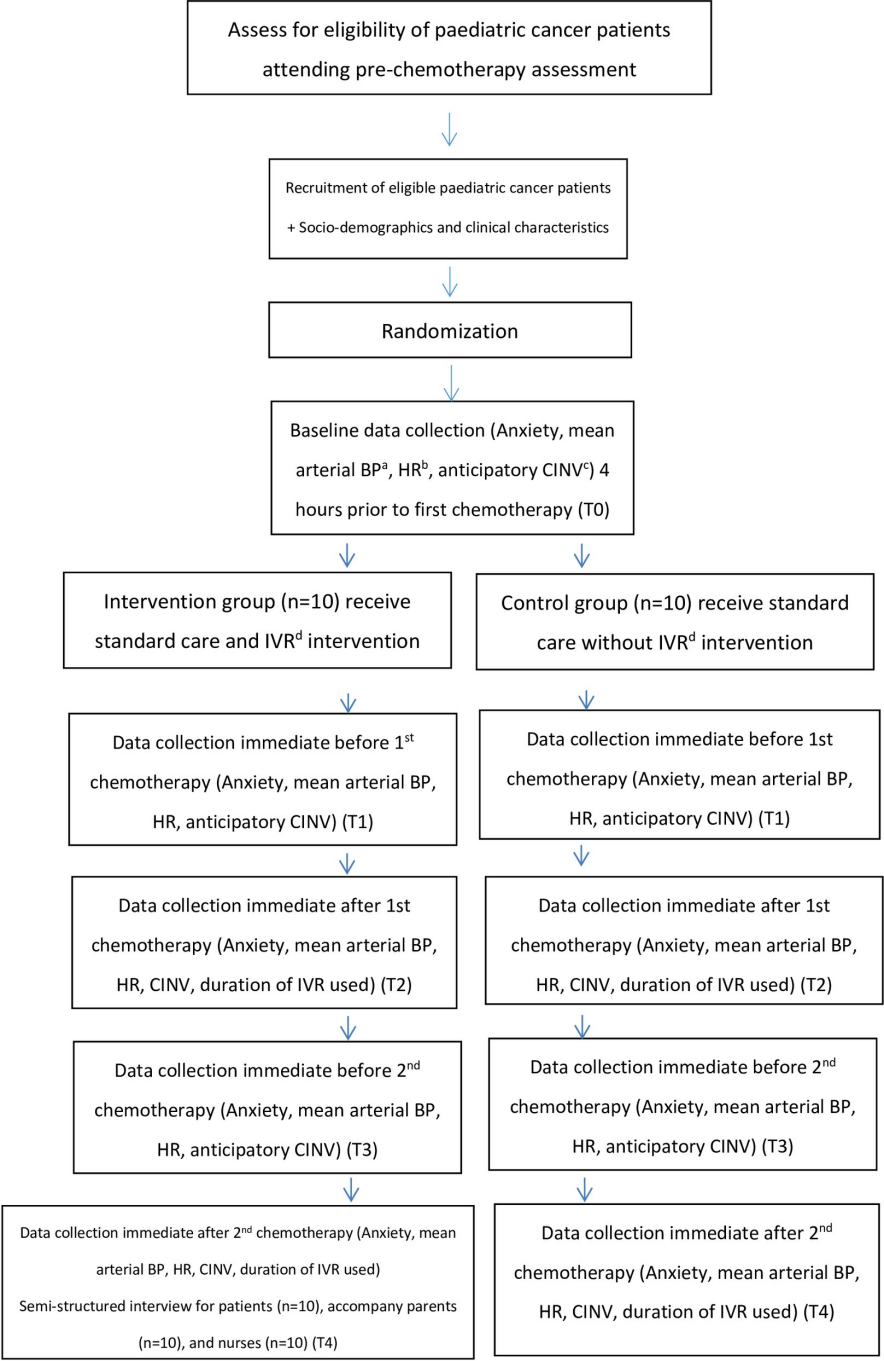
Flow diagram of trial design. ^a^ blood pressure; ^b^ heart rate; ^c^ chemotherapy-induced nausea and vomiting; ^d^ immersive virtual reality.

Individual face-to-face semi-structured interviews will be conducted at T4 with patients in the interventional group, their accompanying parents and oncology nurses involved in the chemotherapy administration. Frequency of vomiting will be obtained from medical record (T0-T4). All children will be given the Google cardboard goggles after the completion of trial. The flow diagram of trial design is shown in Fig 2.

### Data analysis

IBM SPSS for Windows (Version 26) will be used. Appropriate descriptive statistics will be used to summarise data such as screening, eligibility, consent and withdrawal rates.

Continuous demographic (e.g. age) and clinical variables (e.g. scores on CSAS-C) will be presented by their means and standard deviations, whereas categorical data (e.g. sex) will be presented in frequencies and percentages. Pearson’s chi-square test (or Fisher’s exact test) and independent t-test (or Mann-Whitney test for highly skewed data) will be used as appropriate for assessing the homogeneity of the baseline characteristics of the two groups. The intention-to-treat principle will be adopted in the outcome evaluation between the two groups [25]. Generalized estimating equations model will be used to compare each of the outcome measures across the time points between the two groups. GEE model can account for intra-correlated repeated measures data and produce unbiased estimates even in the presence of missing data, provided they are missing at completely random. Potential confounders on the outcome variables, including duration of IVR used, will be adjusted in the GEE models to improve the precision of the effect estimates. Cohen’s d values will also be calculated to estimate the effect sizes of the IVR intervention on the outcome variables. All statistical analyses are two-sided and level of significance will be set at 0.05.

All interviews will be transcribed verbatim into written Chinese before conventional content analysis is conducted. Starting with line-by-line coding, statements that are related to (1) the acceptability and feasibility of the intervention and (2) acceptability and feasibility of trial procedures will be coded and categorized. Once the categories have ample data, they will be broken down into sub-categories. The categories and subcategories will be reviewed by two co-investigators to ensure participants’ points of views are accurately reflected. Appropriate strategies will be undertaken to ensure the trustworthiness including credibility, dependability, confirmability, and transferability [27].

### Reporting guidelines

Standard Protocol Items: We adhered to the Recommendations for Interventional Trials reporting guidelines in this protocol [28].

### Ethics and dissemination

Ethical approval has been sought from the Hong Kong Children’s Hospital Research Ethics Committee with reference number: HKCE-REC-2019-007, on March 1, 2019. Written consent from parents and nurses, as well as assent from children will be obtained prior to study commencement. The findings will be disseminated in peer-reviewed journals and conference presentations.

## Discussion

Pre-chemotherapy anxiety and CINV are the most distressing side effects of paediatric cancer patients undergoing chemotherapy [3, 4]. In view of the negative impacts of unmanaged anxiety and CINV on patents, parents, and medical institutions, effective intervention to manage these symptoms is crucial. However, pharmacological treatments for CINV prevention are still suboptimal [5]. There has been some reports describing distraction and relaxation intervention to addressing the needs of this vulnerable group. However, distractors previously used did not provide complete distraction [9]. IVR allows the patient to be completely engage in an immersive environment, which may help distract the patient from noxious stimuli during chemotherapy, thereby reducing the distressing symptoms.

This exploratory trial aims to examine the acceptability and feasibility of IVR, a novel intervention for distracting paediatric cancer patients undergoing chemotherapy in Hong Kong. Interviews will also be conducted with patients, parents, and healthcare providers to assess satisfaction and any improvements. The mixture of quantitative and qualitative methods is essential to inform the feasibility of recruiting and retaining of patients, the acceptability of parents, parents, and healthcare providers, and to determine appropriate outcome measures that are important for design of definitive trial [27].

The results of this trial will shed light into the use of distraction and relaxation-based IVR intervention to alleviate the psychological and physical sufferings of paediatric cancer patients who require chemotherapy treatments. It will generate empirical evidence on the feasibility of IVR intervention in clinical practice. If feasible and acceptable, IVR may be considered to incorporate into the clinical settings to provide a convenient, attractive and easily applied intervention to distract and relax paediatric patients undergoing chemotherapy virtually at any time and place.

However, several limitations of this trial must be acknowledged. First, only paediatric cancer patients between the ages of 6–12 years will be recruited. Patients under six years of age who are prone to anxiety during chemotherapy will not be included. Therefore, the trial results may not be able to generalise to other age groups. Second, this trial will only be conducted in a single institution so contamination between intervention and control group cannot be ruled out. Nevertheless, the intervention will take place in the patients’ rooms to minimize potential bias. Finally, the trial will only examine the preliminary short-term effects of IVR intervention on anxiety and CINV outcomes (after the second chemotherapy). Therefore, the sustainability of IVR intervention over long periods cannot be determined.

In summary, the proposed IVR intervention will potentially provide insights into the feasibility and acceptability of IVR intervention in managing anxiety, nausea and vomiting among paediatric cancer patients receiving their first chemotherapy. The results will provide a foundation for future studies.

## Trial status

This article presents the protocol for the exploratory trial (original v1). The recruitment period started on May 2019. However, due to COVID-19, recruitment has been suspended since December 2019. At the time of the first version of manuscript submission, data collection was still ongoing.

## Supporting information

S1 Checklist SPIRIT checklist. (DOC)

S1 File Study protocol. (DOCX)
