# Supplementary material for: Effects of immersive virtual reality for preventing and managing anxiety, nausea and vomiting among paediatric cancer patients receiving their first chemotherapy: A study protocol for an exploratory trial
Source: PLoS One. 2021 Oct 14;16(10):e0258514. doi: 10.1371/journal.pone.0258514 (PMC8516310; doi:10.1371/journal.pone.0258514)
Supplement: S1 File — (DOCX) [file pone.0258514.s002.docx]

**S1 File. Study protocol.**

**PROPOSED RESEARCH PROJECT**

|  | Title: | |
| --- | --- | --- |
|  | Effects of immersive virtual reality for preventing and managing anxiety, nausea and vomiting among paediatric cancer patients receiving their first chemotherapy: An exploratory trial | |
|  |  | |
|  | Introduction: | |
|  | **Anxiety and chemotherapy-induced nausea and vomiting**  Chemotherapy treatment can provoke anxiety in many patients.^1^ In particular, patients usually exhibit a heightened level of anxiety when receiving their first chemotherapy.^2^ An observational study found that 89% of chemotherapy-naïve patients experienced pre-chemotherapy anxiety. Paediatric cancer patients exhibiting elevated anxiety level early in the therapy are four times more prone to have elevated anxiety after the treatment.^1^ Moreover, pre-chemotherapy anxiety, as well as anxiety experienced after chemotherapy start, expose patients to a higher risk of chemotherapy-induced nausea and vomiting (CINV).^3, 4^  CINV remains the most frequent and unpleasant side effect in paediatric patients receiving chemotherapy.^5^ Acute CINV refers to symptoms that occur within 24 hours of chemotherapy administration.^5^ Anticipatory CINV may develop without proper initial management. Anticipatory CINV is a conditioned response that occurs prior to a subsequent initiation of chemotherapy upon being re-exposed to the stimuli that signal the chemotherapy infusion.^5^ A meta-analysis of 35 studies reveal that 30% of adult and paediatric patients reported anticipatory CINV.^6^ One known factor associated with CINV development among paediatric patients is emetogenicity of chemotherapy.^7^ However, pharmacological treatments for CINV prevention are still suboptimal.^5^  Poorly managed anxiety and CINV lead to physical consequences, such as dehydration and electrolyte imbalance, and hinder the patients’ ability to cope with subsequent chemotherapy. This phenomenon then leads to the delay or discontinuation of treatment and reduces the survival chances of the patient.^7^ CINV is the most aversive condition that greatly distresses patients and parents, thereby reducing their satisfaction to care.^7^  Effective management of anxiety and CINV is critical for children receiving their first chemotherapy; in particular, anticipatory and acute CINV increase with time.^6^ International guidelines recommend optimal psychological interventions be offered to children for anticipatory CINV.^8^ Therefore, psychological interventions are needed to help paediatric cancer patients to cope with anxiety and CINV on their first chemotherapy.  **Distraction and relaxation for reducing anxiety, nausea and vomiting**  Distraction is among the most effective psychological interventions in reducing anxiety and distress in paediatric patients.^9^ A systematic review and meta-analysis in paediatric patients show that distraction interventions without adult involvement and use of interactive form are more effective in reducing distress, especially for patients younger than 12 years old.^9^  Our previous work revealed that distraction and relaxation are useful for paediatric patients undergoing non-painful medical procedures. For example, the principal investigator (PI) examined the effectiveness of preparation and distraction on reducing anxiety in paediatric patients during cast removal procedures. Results revealed that children in the intervention group showed a statistically significant reduced anxiety levels (p=0.01) compared with those in the control group.^10^ A Co-I (CWH) conducted an exploratory trial in support of the use of nurse-led relaxation exercises and distraction to alleviate anxiety, nausea and vomiting among 20 paediatric cancer patients undergoing chemotherapy.^11^ This trial aimed to produce a relaxing and pleasant response as counter condition before the unpleasant procedure commenced.^11^ However, these face-to-face distractions and relaxation interventions required adult involvement and intensive labour. Therefore, other cost-effective and interactive options should be considered.  **Immersive virtual reality as distraction and relaxation Intervention**  Virtual reality (VR) is an interactive form of distraction wherein a human becomes an active participant in a virtual environment.^12^ VR ranges from non-immersive to fully immersive depending on the degree of the user’s isolation from physical surroundings. In immersive VR (IVR), immersion is created through a head-mounted display which allows 3D interaction between the user and computer. Compared with non-immersive VR, IVR has advantages, including less specialized facilities required and provision of an immersive environment that completely turns the participants’ attention away from the noxious clinical environment.^12^ Distraction-based IVR intervention is already implemented in the Lucile Packard Children’s Hospital at Stanford in the United States.^13^ IVR is also used as distraction and relaxation intervention for patients undergoing chemotherapy in one of two comprehensive cancer centres in Australia.^14^ No adverse effect was reported by the patients in the IVR group.^15^  **Effectiveness of immersive virtual reality for chemotherapy**  A recent review suggested that IVR effectively reduces anxiety and distress for adults and paediatric patients immediately after chemotherapy sessions.^12^ In addition, the efficacy of IVR does not decrease after 8 weeks of repeated exposures in a controlled laboratory environment.^16^ IVR also helped patients to alter their perception of time, thus making the chemotherapy treatment seem short.^17,18^ A pilot study on 11 paediatric patients (aged 10–17) suggested that IVR can be implemented and positively accepted.^19^ Another pre- and post-test study revealed that children receiving chemotherapy reported improved anxiety and distress symptoms, including nausea and vomiting, after one session of IVR intervention.^2^ Nevertheless, these findings are limited by methodological weaknesses, such as a lack of control group or pre-test evaluation or a lack of follow up.^17-19^ In addition, the assessment of paediatric patients, parents and health care providers’ perception and satisfaction towards IVR intervention is seldom included.^12^ | |
|  | **Importance of undertaking this exploratory trial**  To our knowledge, no previous work is conducted to examine the effects of IVR on paediatric cancer patients undergoing chemotherapy in Hong Kong. As such, an innovative study is proposed for the assessment of the effect of IVR to establish relaxation as counter conditioning and distraction in the intervention package. The Medical Research Council presented a framework for the evaluation of a complex intervention and recommended a stepwise approach to evaluation with exploratory trial preceding a full randomized controlled trial.^20^ The proposed exploratory trial with a mixture of quantitative and qualitative method is vital to inform the theoretical design, test the procedures for acceptability, estimate the likely rates of recruitment and retention of subjects and identify appropriate outcome measures for a future full-scale trial.^20^ | |
|  | Study design and objectives | |
|  | An exploratory trial supplemented with qualitative methods. Participants will be randomly allocated to an intervention group that will receive the IVR intervention or the control group. Individual face-to-face semi-structured interviews with patients, parents, and oncology nurses will be conducted to explore the acceptability and feasibility of the intervention. This exploratory trial aims to assess the feasibility and acceptability of IVR as a relaxation and distraction intervention for reducing anxiety and CINV among paediatric cancer patients receiving their first chemotherapy.  The objectives of this trial are:   1. To assess the parameters and feasibility for the design of a definitive trial,   - Screening, eligibility, consent and withdrawal rates.   1. To assess the data collection procedure and collection of preliminary data,   - Anxiety (self-reported and physiological responses including heart rate and mean arterial blood pressure), anticipatory and acute CINV between the intervention and control groups.   1. To assess the satisfaction of the chemotherapy procedure to parents and nurses. 2. To explore ways of improvement for the implementation of intervention and the acceptability of the intervention to patients, parents and nurses. | |
|  | Plan of Investigation: | |
|  |  | Subjects  **Paediatric cancer patients**  Patients and their parents will be recruited from the paediatric oncology unit of a regional public hospital.  Inclusion criteria: Patients who are (1) aged between 6 and 12, (2) chemotherapy naïve, (3) scheduled to receive their first intravenous chemotherapy and (4) can understand Chinese.  Exclusion criteria: Patients who have (1) identified cognitive and learning problems in their medical record, (2) brain tumours or metastasis, (3) identified contact precautions and (4) previous history of seizures or motion sickness.  The rationale for selecting patients aged 6-12 years is because younger patients are more susceptible to anxiety when undergoing medical procedures as compared with older ones. Therefore, their cooperation and response to the chemotherapy procedure and intervention are of concern.^10,11,15^ Meanwhile, this homogenous age group undergoes the same concrete operational developmental stages and thus is more responsive to distraction and relaxation intervention.^9,11^  **Accompanying parents**  All accompany parents will be invited to assess their satisfaction for the chemotherapy procedures. Parents in the intervention group will also be invited to a semi-structured interview to explore their acceptability of the intervention.  **Oncology nurses**  All oncology nurses involved in administration of chemotherapy will be invited to assess their satisfaction for the chemotherapy procedures. Nurses in the intervention group will also be invited to semi-structured interviews to explore their perceptions of trial procedures and the feasibility of integrating the intervention into routine clinical practice.  Sample size  In 2017, about 28 paediatric cancer patients aged between 6 and 12 years were admitted to the study institution for their first chemotherapy. This exploratory trial recruits 20 patients, 20 accompany parents and 10 oncology nurses involved in the administration of chemotherapy. Based on our previous study on paediatric patients with cancer, this sample size can be realistically recruited within the study period and is adequate for the objectives of the trial.^11,15^ |
|  |  | Methods  **Subject allocation and blinding**  This exploratory trial comprises two groups: an intervention group that receives the IVR intervention and standard care and a control group that receives standard care only. Given that different diagnosis require chemotherapy with different emetogencity,^6, 11^ a computer-based stratified randomization is used to randomly assign patients into one group or the other, approach commonly adopted in previous studies. In this categorization, strata are constructed on the basis of cancer types, such as leukaemia, lymphoma and sarcoma etc., which commonly occur in Hong Kong.  The nature of an IVR intervention may be impractical for the blinding of participants and outcome assessors. Nevertheless, the lack of blinding does not necessarily contribute to a source of bias because children are unlikely to change their behaviours even when they are aware of participating in a certain intervention.^10, 11^  **Control: standard care**  Participants in the control group will receive standard care only with no IVR intervention during their chemotherapy. Standard care consists of pre-chemotherapy instruction and intravenous antiemetic administration as needed.  **Intervention: IVR**  Participants receive IVR for three separate sessions in the first and second course of chemotherapy: (1) 4 hours prior to the chemotherapy commenced, (2) 5 minutes before and during the first chemotherapy and (3) 5 minutes before and during the second chemotherapy.  Considering that unconditioned stimulus–conditioned stimulus pairings are sufficient even for one course of chemotherapy exposure, conducting additional sessions may result in missing data despite enhancing the intervention effect. Therefore, three sessions of IVR that involve the first and second course of chemotherapy is feasible.^21^  The first IVR session is used to establish a relaxation and playful response as counter conditioning before the chemotherapy commenced. As patients are usually admitted in the morning to prepare for chemotherapy infusion that is commenced in the afternoon, the first IVR session is held 4 hours before commence of chemotherapy for practical reason.  The second IVR session is used to establish IVR as counter conditioning and distraction intervention during chemotherapy infusion to reduce the anxiety and CINV of patients.  The third session aims to determine if IVR has stable effects on reducing anxiety and CINV, and whether or not patients can sustain their interest in IVR.  During the intervention, the patients experience the sense of immersion through a head-mounted device delivering IVR sounds and images by using VR goggles that can be fitted to both Apple and Samsung smartphones.  Our previous work on IVR among Hong Kong paediatric cancer patients found that 3D cartoon videos, such as Minions or Cut the rope, are preferred by patients aged below 12; however, VR sceneries are not selected by any children. As such, four 3D cartoon modules, which provide a wide range of visual and auditory stimuli and can be freely downloaded, are provided for the patients. These videos are also interesting and captures the attention of patients for an extended period of time.^15^    **Implementation protocol**  A trained research assistant (RA) provides the patients with standard instruction on how to use the IVR equipment in the first session. The VR goggles will then be placed and adjusted on the patient’s head to ensure comfort and secure fit. For the first session, patients are given 30 minutes to use and familiarise themselves with the IVR equipment.^17-18^  For the second and third sessions, the RA provides IVR to patients 5 minutes before the chemotherapy commences.^2,19^ The RA asks the oncology nurse to start the administration once the patients are ready for the chemotherapy treatment. The nurse first disinfects outlets of the intravenous site, connect the device and start infusion, during which patients will be allowed to view their selected VR modules or take off the headset at will. The RA will record the time of each module used by the patients by using a time-counting device. When the infusion is over, the RA will remove the IVR equipment.  **Fidelity of the intervention**  The fidelity of the intervention is ensured by recruiting a part-time research assistant (RA) who has at least two years of experience in paediatric care. The investigator will deliver 1.5 days of training to RA in (1) evidence of the benefits of IVR to paediatric patients; (2) common chemotherapy regimens; (3) procedures of implementing the intervention; and (4) application and basic knowledge about IVR.  At least one session conducted by the RA each month is randomly selected to assess compliance with the implementation protocol by the principal investigator.  **Measurements**  **Objective 1: parameters and feasibility for designing a definitive trial**  The following data will be collected, the definitions of each type being as follows.  ***Screening rate***  The number of patients admitted to the paediatric oncology unit and screened for eligibility by the oncology nurse.  ***Eligibility rate***  The number of patients fulfilling the inclusion criteria divided by the number of patients admitted to the unit for their first chemotherapy.  ***Consent rate***  The number of patients whose parents give consent divided by the number of patients eligible for the trial. Those who do not give consent will have their reasons for declining (provided in voluntary basis) as recorded by the RA.  ***Withdrawal rate***  The withdrawal rate refers to the number of patients withdrawing from the trial after giving consent. For ethical reasons, the number of participants who decline to participate in the project will only be recorded instead of asking them for the reason of their refusal to participate. However, such data will be recorded down if the patients provided in voluntary basis.  **Objective 2: measurement of outcomes to collect preliminary data**  The following outcome measures will be collected by the research assistant.  ***Anxiety***  ***The short form of the Chinese version of the State Anxiety Scale for Children (CSAS-C)***  The CSAS-C will be used to measure the anxiety levels of the participants in the trial (Appendix 1).^22^ This measurement uses a three-point Likert scale with 10 items and total scores ranging from 10 to 30, with higher scores indicating higher anxiety levels.^22^ The State Anxiety Scale has been used in our previous IVR study to measure anxiety among children undergoing needle-related procedures, with a Cronbach’s alpha coefficient of 0.89.^15^    ***Heart rate and mean arterial blood pressure***  The physiological responses of anxiety will be measured by heart rate and mean arterial blood pressure (HR & BP) using a standard automatic blood pressure monitoring machine (available in the study institution). These indicators are considered to be objective and definitive in assessing physiological responses of anxiety.^10,11^    ***Anticipatory nausea and vomiting***  A visual analogue scale (VAS) will be used to assess the severity of nausea and vomiting before the chemotherapy^4^ (Appendix 2). Children will be asked to indicate their severity of nausea and vomiting on a 0–100 mm horizontal line, with “0” indicating the absence of nausea and vomiting while “100” indicates the most severe form of nausea and vomiting.  ***Acute nausea and vomiting***  The MASCC Antiemesis Tool (MAT) will be adopted to assess the chemotherapy-induced nausea and vomiting. The Chinese version has good reliability and validity with a Cronbach’s alpha coefficient of 0.73.^23^ The four items assessing acute CINV will be used in this trial (Appendix 3).  **Objective 3: satisfaction of the chemotherapy procedure to parents and nurses**  The parent and nurse satisfaction levels toward the chemotherapy procedure will be assessed by questionnaires developed by Tyson and colleagues (2014). The original English questionnaire for parents is a 10-item scale rated on a five-point scale ranging from 1 = strongly disagree to 5 = strongly agree. Higher scores indicate higher levels of satisfaction (Appendix 4).  The satisfaction of the nurses will be examined by eight items, with each being rated on a scale from 1 = strongly disagree to 5 = strongly agree (Appendix 5).  The PI have translated both questionnaires into Chinese using the back-translation method recommended by Brislin (1986). The translated version was reviewed by a panel of expert professionals for semantic and content equivalence. This scale has been used in previous study to assess the satisfaction level of parents and health care professionals toward a medical procedure with the reported Cronbach’s alpha 0.90 for both scales.^10^  **Objective 4: acceptability of the intervention**  Individual face-to-face semi-structured interviews will be conducted using an interview guide (Appendix 6) with 10 patients, their accompanying parents and the nurses who administrate chemotherapy to the intervention group.  The interviews with the patients aim to explore their experiences, acceptance, satisfaction and any improvements of the IVR intervention.  The interviews with parents aim to explore their acceptance and possible improvements in implementing IVR for their child.  The interviews with nurses aim to determine the feasibility of integrating IVR intervention into their routine clinical practice and any possible improvements. The RA, who also has considerable experience of qualitative interviews, will conduct the interviews. Each interview will be audio-taped and conducted in a quiet room provided at the study institution.    Socio-demographic and clinical characteristics  The following clinical characteristics will be collected from participant's medical record: age, gender, year of study, medical diagnosis, time since diagnosis, stage of disease, type of cancer treatment received, chemotherapy regimen, types and dosage of antiemetic prescribed and intake (Appendix 7). These will be useful in providing baseline information for optimizing design and subject recruitment for the future definitive trial.  **Procedures**  A nurse in the paediatric oncology unit will screen for eligibility for all patients who are admitted to receive their first intravenous chemotherapy during pre-chemotherapy assessment. If children meet the inclusion criteria, the nurse will refer them and their accompanying parents to the RA who will give them an information sheet, explain the trial and show the IVR equipment. If they agree to participate, an informed consent will be obtained from the accompanying parents. The RA will then acquire socio-demographics and clinical characteristics of the patients from medical record. According to the subject allocation, children in the control group will receive standard care, while the intervention group will additionally receive the IVR intervention.  Four hours prior to (T0), immediate before first course (T1), and second course of chemotherapy begins (T3), a set of data (i.e. anxiety, HR & BP and anticipatory CINV) will be collected from the patients.  Immediate after the first (T2) and second course of chemotherapy (T4), the anxiety, HR & BP and acute CINV will be collected. In the intervention group, the timing and duration of IVR use and modules selected will also be recorded.  Individual face-to-face semi-structured interviews will be conducted at T4 with patients in the interventional group, their accompanying parents and oncology nurses involved in the chemotherapy administration. Frequency of vomiting will be obtained from medical record (T0-T4). All children will be given the VR goggles after the completion of trial.  **Ethical conduct**  Ethical approval is obtaining from the Ethical Committees of the study institutions. The purpose and details of the trial will be clearly provided before obtaining parental consent. The confidentiality and anonymity of any data collected will be assured. |
|  |  |  |
|  |  |  |
|  |  | Data processing and analysis  **For objectives 1 to 3**  IBM SPSS for Windows (Version 24) will be used. Appropriate descriptive statistics will be used to summarise data such as screening, eligibility, consent and withdrawal rates.  Continuous demographic (eg. age) and clinical variables (eg. scores on CSAS-C) will be presented by their means and standard deviations, whereas categorical data (eg. sex) will be presented in frequencies and percentages. Pearson’s chi-square test (or Fisher’s exact test) and independent t-test (or Mann-Whitney test for highly skewed data) will be used as appropriate for assessing the homogeneity of the baseline characteristics of the two groups. The intention-to-treat principle will be adopted in the outcome evaluation between the two groups.^24^ Generalized estimating equations model will be used to compare each of the outcome measures across the time points between the two groups. GEE model can account for intra-correlated repeated measures data and produce unbiased estimates even in the presence of missing data, provided they are missing at completely random. Potential confounders on the outcome variables, including duration of IVR used, will be adjusted in the GEE models to improve the precision of the effect estimates. Cohen’s d values will also be calculated to estimate the effect sizes of the IVR intervention on the outcome variables. All statistical analyses are two-sided and level of significance will be set at 0.05.  **For objective 4**  All interviews will be transcribed verbatim into written Chinese before conventional content analysis is conducted. Starting with line-by-line coding, statements that are related to (1) the acceptability and feasibility of the intervention and (2) acceptability and feasibility of trial procedures will be coded and categorized. Once the categories have ample data, they will be broken down into sub-categories. The categories and subcategories will be reviewed by two co-investigators to ensure participants’ points of views are accurately reflected. Appropriate strategies will be undertaken to ensure the trustworthiness of the study including credibility, dependability, confirmability, and transferability.^24^ |
|  |  |  |
|  |  | |
|  |  | |
|  | Key References: | |
|  | 1. Carnio S, Galetta D, Scotti V, Cortinovis DL, Antonuzzo A, Pisconti S, … Novello S. Chemotherapy-induced nausea and vomiting (CINV) in patients with advanced lung cancer during the first-line treatment: assessment by physicians, nurses, and patients from an Italian multicentre survey. Support Care Cancer 2017. doi:10.1007/s00520-017-4004-1. 2. Schneider SM, Workman ML. Effects of virtual reality on symptom distress in children receiving chemotherapy. Cyberpsycho Behav 1999;2(2):125-34. 3. Chan A, Kim HK, Hsieh RK, Yu S, De Lima LG, Su WC, Banos A, Bhatia S, Burke TA, Keefe DMK. Incidence and predictors of anticipatory nausea and vomiting in Asia Pacific clinical practice-a longitudinal analysis. Supportive Care Cancer 2015;23(1):283-91. 4. Molassiotis A, Lee PH, Burke TA, Dicato M, Gascon P, Roila F, Aapro M. Anticipatory nausea, risk factors, and its impact on chemotherapy-induced nausea and vomiting: results from the Pan European Emesis Registry Study. J Pain Symptom Manage 2016;51(6):987-93. 5. Dupuis LL, Sung L, Molassiotis A, Orsey AD, Tissing W, wan de Watering M. 2016 updated MASCC/ESMO consensus recommendations: Prevention of acute chemotherapy-induced nausea and vomiting in children. Support Care Cancer. 2017;25(1):323-31. 6. Morrow GR, Roscoe JA, Kirshner JJ, Hynes HE, Rosenbluth RJ. Anticipatory nausea and vomiting in the era of 5-HT3 antiemetics. Support Care Cancer 1998;6:244-247. 7. Vol H, Flank J, Lavoratore SR, Nathan PC, Taylor T, Zelunka E, Maloney AM, Lee Dupuis L. Poor chemotherapy-induced nausea and vomiting control in children receiving intermediate or high dose methotrexate. Support Care Cancer 2016;2(3):1365-71. 8. Dupuis LL, Robinson PD, Boodhan S, Holdsworth M, Portwine C, Gibson P, Philips R, Maan C, Stefin N, Sung L; Pediatric Oncology Group of Ontario. Guideline for the prevention and treatment of anticipatory nausea and vomiting due to chemotherapy in pediatric cancer patients. Pediatr Blood Cancer 2015;61(8):1506-12. 9. Birnie KA, Noel M, Parker JA, Chambers CT, Uman LS, Kisely SR, McGrath PJ. Systematic review and meta-analysis of distraction and hypnosis for needle-related pain and distress in children and adolescents. J Pediatr Psychol 2014;39(8):783-808. 10. Wong CL. The best partner in a stressful medical procedure: Play. Paper presented at the Imagining the Future: Community Innovation and Social Resilience in Asia, The Chinese University of Hong Kong, Hong Kong (2017, February). 11. Carmen CWH, Lam LW, Li CK, Cheung JS, Cheng KKF, Chik KW, Tang WPY. Feasibility of psychoeducational intervention in managing chemotherapy-associated nausea and vomiting (CANV) in pediatric oncology patients. Eur J Oncol Nurs 2015;19(2):182-90. 12. Chirico A, Lucidi F, De Laurentiis MD, Milanese C, Napoli A, Giordano A. Virtual reality in health system: Beyond entertainment. A mini-review on the efficacy of VR during cancer treatment. J Cell Physiol 2016;231(2):275-287. 13. Stanford Children’s Health. Hospital-wide access to virtual reality alleviates pain and anxiety for pediatric patients. Available from http://www.stanfordchildrens.org/en/about/news/releases/2017/virtual-reality-alleviates-pain-anxiety?source=whats-new. [Accessed 6th February 2018]. 14. Samsung Newsroom. Start VR introduces virtual reality to chemotherapy patient program at Chris O’Brien Lifehouse. Available from <https://news.samsung.com/global/start-vr-introduces-virtual-reality-to-chemotherapy-patient-program-at-chris-obrien-lifehouse>. [Accessed 6^th^ February 2018]. 15. Yeung MT, Wong CL, Chan ON. (accepted). Effects of immersive virtual reality on anxiety among paediatric cancer patients undergoing peripheral intravenous cannulation: preliminary results of a pilot study. Paper presented at the 8^th^ Nursing Symposium on Cancer Care, Hong Kong. 16. Rutter CE, Dahlquist LM, Weiss KE. Sustained efficacy of virtual reality distraction. J Pain 2009;10(4):391-7. 17. Schneider SM, Hood LE. Virtual reality: a distraction intervention for chemotherapy. Oncol Nurs Forum 2007;34(1):39-46. 18. Schneider SM, Kisby CK, Flint EP. Effect of virtual reality on time perception in patients receiving chemotherapy. Support Care Cancer 2011;19(4):555-64. 19. Schneider SM, Workman ML. Virtual reality as a distraction intervention for older children receiving chemotherapy. Pediatr Nurs 2000;26(6):593-7. 20. Craig P, Dieppe P, Macintyre S, Nazareth I, Petticrew M: Developing and evaluating complex interventions: the new Medical Research Council guidance. Br Med J 2008;337:a1655. 21. Geiger F, Wolfgram L. Overshadowing as prevention of anticipatory nausea and vomiting in pediatric cancer patients: study protocol for a randomized controlled trial. Trials 2013;14:103. 22. Li HCW, Lopez V. Development and validation of a short form of the Chinese version of the State Anxiety Scale for Children. Int J Nurs Stud 2007;44(4):566-73. 23. Tan JY, Suen LK, Molassiotis A. Psychometric assessment of the Chinese version of the MASCC Antiemesis Tool (MAT) for measuring chemotherapy-induced nausea and vomiting. Support Care Cancer 2016;24(9):3729-37. 24. Polit DF, Beck CT. Essentials of nursing research: Appraising evidence for nursing practice. 8th ed. Philadelphia: Lippincott Williams & Wilkins; 2013. | |
